# Evaluation of a novel cardiac signal processing system for electrophysiology procedures: The PURE EP 2.0 study

**DOI:** 10.1111/jce.15250

**Published:** 2021-10-01

**Authors:** Amin Al‐Ahmad, Bradley Knight, Wendy Tzou, Robert Schaller, Omar Yasin, Deepak Padmanabhan, Jason Zagrodzky, Mohammed Bassiouny, J. David Burkhardt, G. Joseph Gallinghouse, Moussa Mansour, Christopher McLeod, Andrea Natale

**Affiliations:** ^1^ Texas Cardiac Arrhythmia Institute Austin Texas USA; ^2^ Electrophysiology Section, Division of Cardiology Northwestern University Medical Center Chicago Illinois USA; ^3^ Electrophysiology Section, Division of Cardiology University of Colorado Denver Colorado USA; ^4^ Electrophysiology Section, Division of Cardiology Hospital of the University of Pennsylvania Philadelphia Pennsylvania USA; ^5^ Electrophysiology Section, Division of Cardiology Mayo Clinic Rochester Minnesota USA; ^6^ Sri Jayadeva Institute of Cardiovascular Science and Research Bengaluru India; ^7^ Electrophysiology Section, Division of Cardiology Massachusetts General Hospital Boston Massachusetts USA

**Keywords:** intracardiac signals, signal acquisition, signal processing, signals

## Abstract

**Background:**

Intracardiac electrogram data remain one of the primary diagnostic inputs guiding complex ablation procedures. However, the technology to collect, process, and display intracardiac signals has known shortcomings and has not advanced in several decades.

**Objective:**

The purpose of this study was to evaluate a new signal processing platform, the PURE EP™ system (PURE), in a multi‐center, prospective study.

**Methods:**

Intracardiac signal data of clinical interest were collected from 51 patients undergoing ablation procedures with PURE, the signal recording system, and the 3D mapping system at the same time stamps. The samples were randomized and subjected to blinded, controlled evaluation by three independent electrophysiologists to determine the overall quality and clinical utility of PURE signals when compared to conventional sources. Each reviewer assessed the same (92) signal sample sets and responded to (235) questions using a 10‐point rating scale. If two or more reviewers rated the PURE signal higher than the control, it was deemed superior.

**Results:**

A total of 93% of question responses showed consensus amongst the blinded reviewers. Based on the ratings for each pair of signals, a cumulative total of 164 PURE signals out of 218 (75.2%) were statistically rated as Superior for this data set (*p* < .001). Only 14 PURE signals out of 218 were rated as Inferior (6.4%).

**Conclusion:**

The PURE intracardiac signals were statistically rated as superior when compared to conventional systems.

## 1 INTRODUCTION

Accurate interpretation of intracardiac electrograms remains essential to the field of electrophysiology (EP). Prior studies have described the pathological significance of specific signal characteristics.^1^, ^2^, ^3^, ^4^, ^5^ Unfortunately, the technology to collect, process, and display intracardiac signals has remained relatively unchanged for several decades as other important EP technologies have evolved. The modern EP Lab is complex with advanced mapping systems, high density multi‐electrode diagnostic catheters, and irrigated, contact force sensing ablation catheters. These advancements have allowed electrophysiologists to safely treat the most complex arrhythmias, but room remains to improve the efficiency and outcomes of some ablation procedures.^6^, ^7^

Technological advancements within the EP lab have increased the environmental noise profile which requires additional filtering and gaining of intracardiac signals that results in loss or distortion of important signal data at the highest frequencies and lowest amplitudes.^8^, ^9^ Ultimately, for electrophysiologists to fully describe and understand the most complex arrhythmias, the signal processing technology should be designed to preserve the integrity of all physiologic intracardiac signals regardless of frequency, amplitude, or environmental noise. Recently, the PURE EP™ system (BioSig Technologies) was introduced for this purpose and showed encouraging results in animal models.^10^ This multi‐center, prospective study evaluated the overall quality and clinical relevance of PURE signals when compared to signals from conventional technology.

## 2 METHODS

The study protocol was approved by the Institutional Committee on Human Research at the authors' participating institutions. All patients provided written informed consent. Patients scheduled for elective ablation procedures at either St. David's Medical Center in Austin, TX or the Mayo Clinic in Jacksonville, FL were screened, consented, and enrolled in the study from November 2019 to October 2020. Patients with all types of elective ablation procedures were included: supraventricular tachycardia, atrial fibrillation, atrial flutter, ventricular tachycardia, and premature ventricular contractions (PVC). Those patients with significant congenital anomalies, cardiac surgery within the last 60 days, or active illness/systemic infection were excluded. Surface electrocardiographic and intracardiac signal data of clinical interest were collected during the ablation procedures from PURE, the signal recording system, and the 3D mapping system at the same timestamps. Collected signals underwent core lab review and signals of clinical interest were annotated. To minimize bias, the signal samples were then randomized and subjected to blinded, controlled evaluation by three independent electrophysiologists to determine the overall quality and clinical utility of PURE signals when compared to conventional sources. Each blinded reviewer responded to questions using a 10‐point rating scale. The assessment questions fell into the following categories: (a) small, fractionated signals of clinical interest, (b) confidence in discerning near‐field (NF) versus far‐field (FF) signals, (c) ability to interpret signals post‐pacing or post‐CV (d) ability to interpret signals on the Ablation distal (Abld) channel during RF, and (e) overall quality of the signals.

### 2.1 Equipment used and configuration

The PURE system consists of a Main System Unit for acquiring the analog signals and converting the raw data into a digital format, a Main Processing Unit for the analysis and display of the information, (2) junction boxes for connection to various catheter inputs, stackable jumper connection cables, a surface ECG cable, and several monitors and a keyboard. PURE was connected in conjunction with the 3D mapping systems with its own real‐time and review display screens. Stacking jumper cables and junction boxes were utilized to feed signal data from the diagnostic and ablation catheters to PURE. Either the Biosense Webster CARTO®3 or Abbott Precision™ 3D Mapping system was used in combination with the GE CardioLab™ Recording system. Of note, prior testing had been conducted to demonstrate that parallel connections of PURE to conventional systems using stacking jumper cables does not degrade the quality of the signals going to either system. PURE was configured to display the same channels as the recording system. The diagnostic and ablation catheters used were per the physician discretion. The recording and mapping system display/filter settings were optimized per physician preference and normal workflow. Of note, in some procedures a High Pass 50 Hz filter was applied to the AbL D and AbL P channels on PURE, while a 30 Hz filter was routinely applied to the same signals using the GE CardioLab™ system. Lastly, signals collected and submitted for blinded assessment were all bipolar signals.

### 2.2 Signal sample collection process

**Figure 1:**
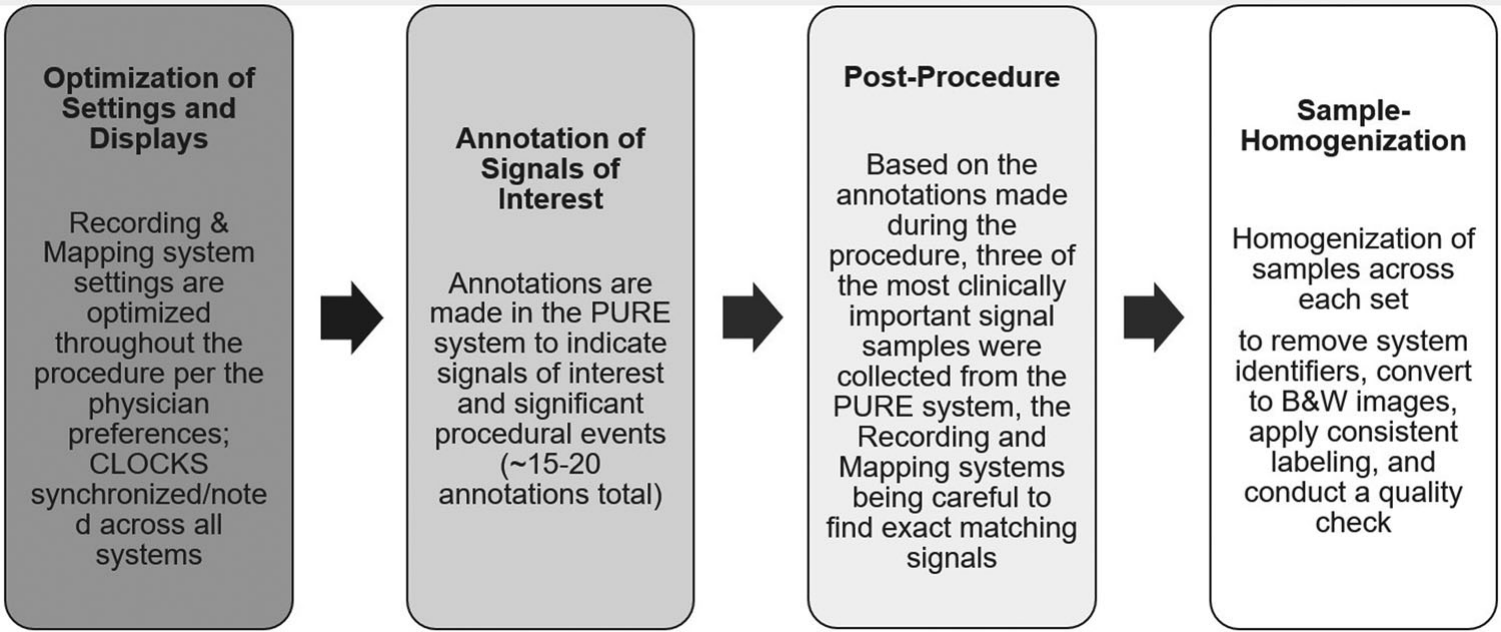
Signal sample collection process

The clock timestamps were noted on each system at the start of each procedure. Throughout the procedure, signals of clinical interest were annotated within PURE utilizing a created annotation library. The use of the annotation library helped create consistency across all the data collected during the study. In an average procedure, there were ~15–20 annotations made. Postprocedure, the annotations were reviewed, and three signal samples were selected. Those (3) PURE signal images were extracted, and then exact matching signals were located on both the mapping and recording systems using the timestamp to ensure that they are the same. Each final sample set contained three image files saved as one document. During the final step, the images went through a homogeneous process to remove system identifiers, convert the images to black and white, confirm consistent labeling, and conduct a quality check (Figure 1).

### 2.3 Core lab and randomization process

**Figure 2:**
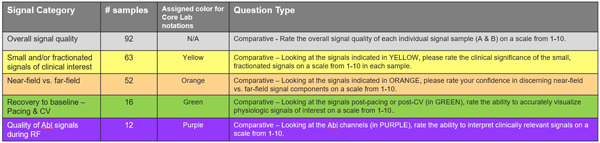
Core lab signal categories, assigned color for notation, and associated assessment question

**Figure 3:**
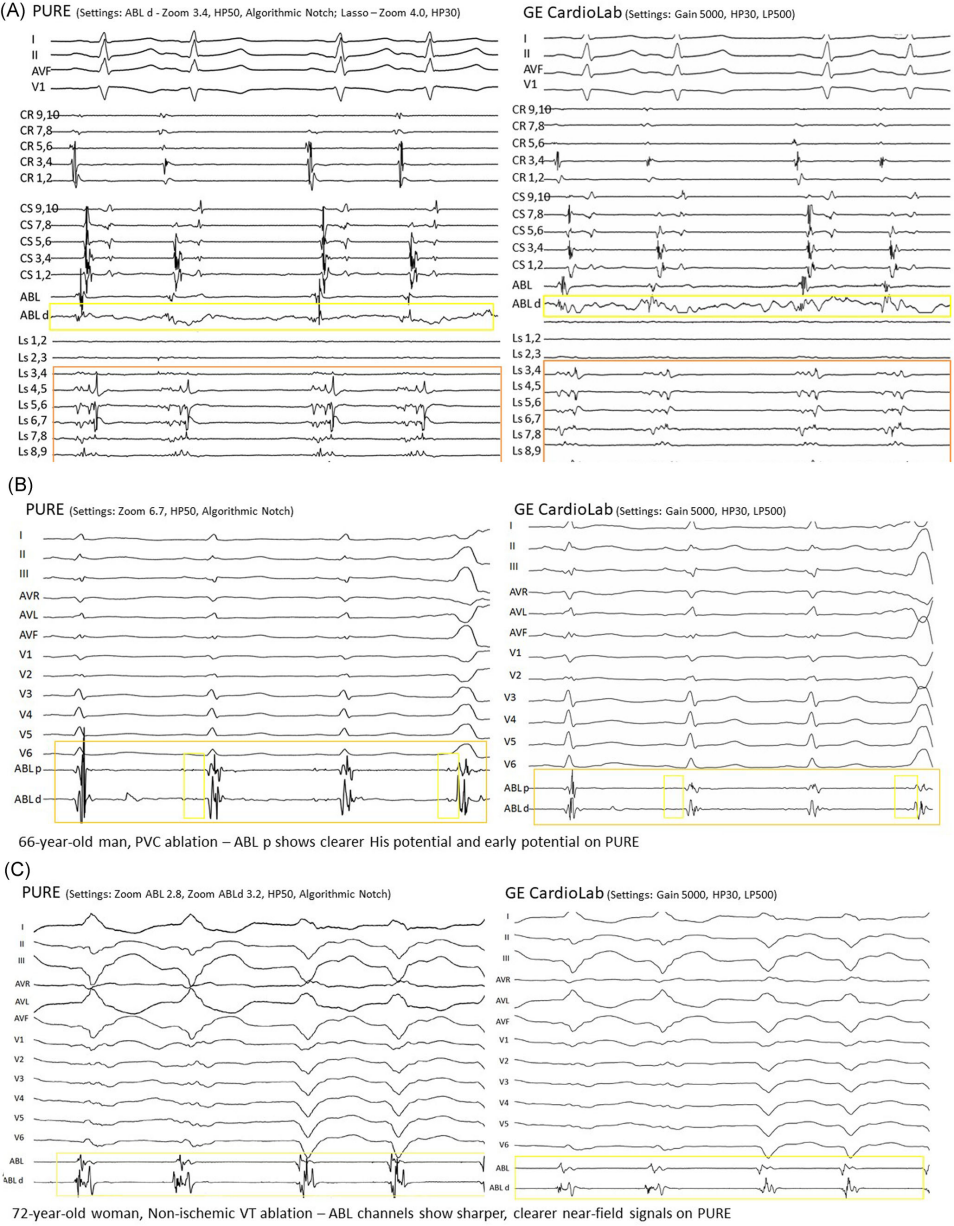
(A) 76‐year‐old man, Persistent AF—PURE shows less noise on ABL d channel and sharper NF signals on Ls channels. (B) 66‐year‐old man, PVC ablation—ABL p shows clearer His and early potential on PURE. (C) 72‐year‐old woman, nonischemic VT ablation—ABL channels show sharper and larger near‐field signals on PURE. PVC, premature ventricular contractions

After all sample sets were collected, two unblinded EP reviewers (D. P. and O. Y.) conducted an expert quality check of each signal sample set and identified specific categories of signals on each figure and highlighted using color‐coded notations (Figure 2). These color‐coded signals became the focus of the blinded assessment. After the core lab review was completed, the sample sets underwent a final review and edits by the Principal Investigator and then two randomizations. The first randomization determined whether the PURE sample would be paired with either the recording or mapping system signal sample. The second randomization determined whether the PURE sample would be on the right or left side of the page. The paired signal samples were arranged side by side on one page and labeled A (left side of the page) or B (right side of the page) (Figure 3). Any sample sets that had mismatched/missing data or were deemed not clinically significant by unblinded EP reviewers were removed from the final assessment.

### 2.4 Blinded assessment

Three independent electrophysiologists participated in the blinded, controlled assessment: B. K., W. T., and R. S. The blinded reviewers were provided basic instructions, a PDF document with the signal sample sets, and a link to an associated electronic data collection tool.

### 2.5 Statistical analysis

Each paired signal rating was collected and unblinded for the analysis after the blinded assessment was completed. Using a binary approach, each signal set was categorized as Superior, Equivalent, or Inferior for PURE. If at least two blinded reviewers rated the PURE signal higher than the control, it was deemed superior. Alternately, if the PURE signal was rated lower than the control by at least two blinded reviewers, it was deemed inferior. If a result showed nonconsensus across the three reviewers, the sample was censored from primary analysis. To determine the statistical significance between superior versus inferior results, Fisher's Exact test was utilized based on the observed rates.

## 3 RESULTS

### 3.1 Assessment responses

**Figure 4:**
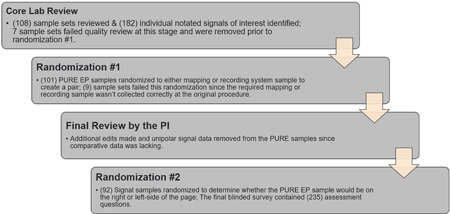
# of samples reviewed and randomized

A total of 108 sample sets from 51 patient enrollments underwent core lab review. Sixteen sample sets failed final quality review and were removed. The reasons for removal are listed in supplement A. The final blinded signal assessment consisted of 92 signal sample sets from 37 patient procedures and contained 235 questions (Figure 4).

**Figure 5:**
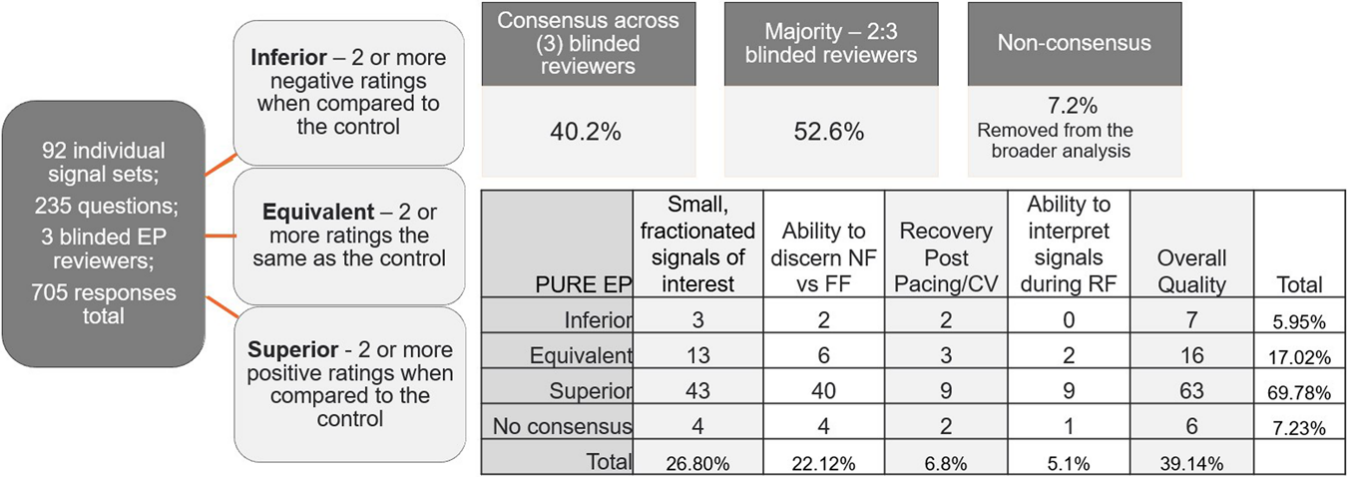
Results of the PURE EP study

A total of 93% of question responses showed consensus across the blinded reviewers. Based on the ratings for each pair of signals, a cumulative total of 164 PURE signals out of 218 (75.2%) were rated as superior for this data set. 14 PURE signals out of 218 were rated as Inferior (6.4%) (Figure 5).

**Figure 6:**
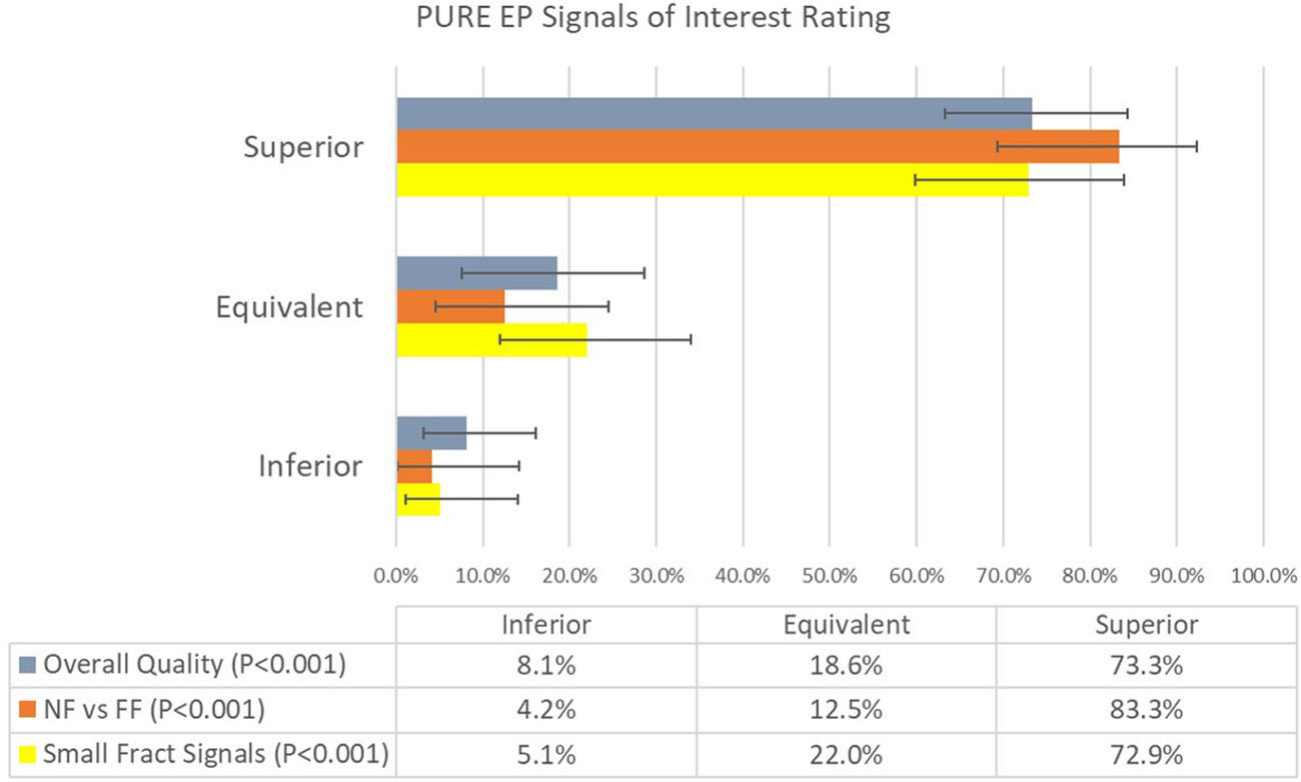
PURE signals were rated statistically better in (3) signal categories: overall signal quality, ability to discern near‐field from far‐field signal components, and clinical value of small/fractionated signals

PURE signals were statistically rated better for overall signal quality when compared to conventional sources 73% of the time (*p* < .001). The blinded reviewers were more confident in discerning NF versus FF signal components on PURE when compared to conventional systems 83% of the time (*p* < .001). The PURE system produced superior small, fractionated signals of clinical significance 73% of the time (*p* < .001) (Figure 6). We found a similar trend in the samples that evaluated recovery to baseline after pacing or cardioversion, as well as the samples for signal quality during RF, however, the number of samples in these two categories were too small for statistical analysis.

**Figure 7:**
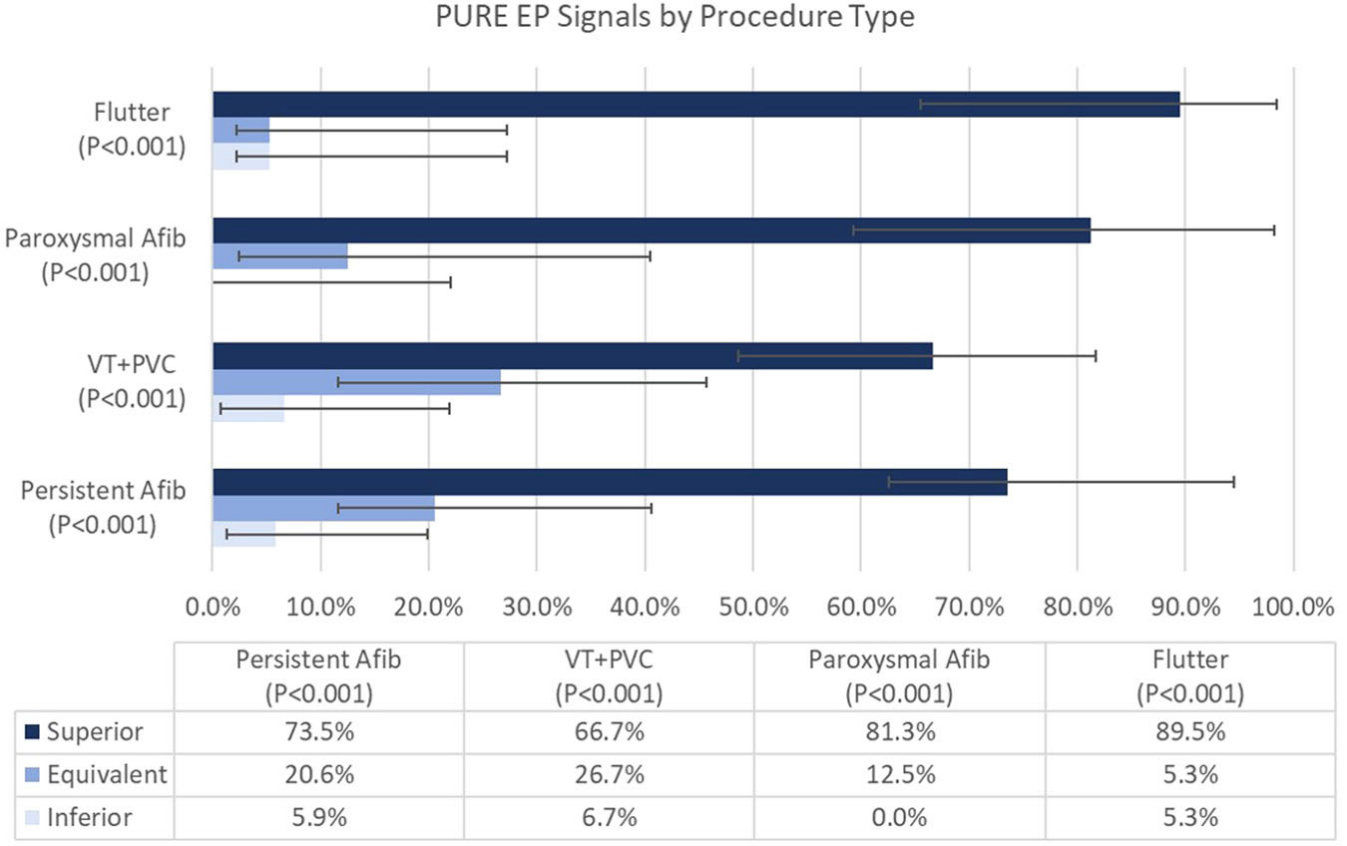
PURE signals were rated statistically better regardless of the type of ablation procedure

Furthermore, across all types of ablation procedures, clinically important intracardiac signals acquired by the PURE system were statistically rated better than matching signals from conventional systems (*p* < .001) (Figure 7). In Persistent AF, Paroxysmal AF, PVC, VT, and Atypical Aflutter PURE signals were rated superior 74%, 81%, 67%, and 90% of the time, respectively.

Lastly, across the data set, a variety of diagnostic, mapping, and ablation catheters with different electrode spacing were used. The signal ratings followed the same statistical pattern regardless of the catheters used, although no in‐depth analysis was performed by specific catheter type or electrode spacing.

## 4 DISCUSSION

To date, there are no study data comparing signal quality across different systems in the EP lab. In this study, we have shown the addition of PURE resulted in superior or equivalent electrogram quality 87% of the time. In many cases, the signal quality of the PURE demonstrated clear and significant areas to potentially to target for ablation beyond the standard recording and mapping system signals. Importantly PURE demonstrated clear superiority across all ablation procedure types included in the study.

Despite the advances in 3D mapping systems and imaging technology, intracardiac signal data remains the most important parameter when therapeutic decisions are made regarding ablation targets.^11^ One of the most significant advances in mapping has been the utility of multipolar electrodes and rapid automated mapping features.^12^ However, inaccurate data related to poor electrogram quality may have a significant impact on the accuracy of the 3D map and the success of the procedure. Accuracy of the metrics of an electro‐anatomical map depends on the fidelity of the recorded signals. Areas of interest on the map may need verification on a second high fidelity system to ensure that shortcomings of signal recording related to electrode size, orientation, and system noise are minimized. This is also critically important in re‐do procedures where prior ablation has impacted the amplitude and quality of intracardiac signals such that they can be difficult to detect using standard signal acquisition systems. In these cases, success of the procedure and improved clinical outcome for the patient may hinge on identification of low amplitude and fractionated electrograms.

The PURE EP™ System used in this study was uniquely designed to address long‐standing limitations in signal quality, such as environmental lab noise, signal saturation, slow signal recovery, and inaccurate display of fractionated potentials. The system brings together a range of improved capabilities to preserve and accurately display physiologic signals: proprietary low‐noise architecture, larger dynamic range, higher frequency bandwidth, and an innovative approach to acquiring unipolar signals. PURE also introduces innovative software capabilities like digital zoom (vs. gain), which enhances important physiologic details while preserving the high signal‐to‐noise ratio, and algorithmic notch, which dynamically eliminates recurring noise patterns.

In the future this technology may also be useful to evaluate important and additional aspects of intracardiac signals. Some examples include a better understanding of NF versus FF signals, an improved accuracy of PPI measurements without problems related to signal saturation, and improved understanding of the His Purkinje system signals that can be important in ablation procedures and novel pacing systems. In addition, evaluation of bipolar and unipolar signal changes during ablation could help assess adequate therapy delivery. Lastly, superior signal data can be used in development and advancement of artificial intelligence algorithms to assist in diagnosis and procedural guidance.

Electrogram assessment using PURE is superior to conventional signal acquisition systems (recording systems as well as electro‐anatomical mapping systems). Further research will be useful to determine if these superior signals will lead to improved outcomes or increased understanding of arrhythmia mechanisms.

### 4.1 Limitations

The method used in this study represents real‐world experience. As such, the conventional systems were optimized per the individual lab environment and physician preference. The control signal images included in this study were captured as displayed during the actual procedure. Therefore, the filter and gain settings on the mapping and recording systems were not specifically prescribed and there was operator‐to‐operator variability across the procedures. There were minor differences in filter settings between PURE and the conventional systems in this study. A separate internal evaluation of the PURE samples with identical conventional filter settings applied did not result in discernable degradation in quality or other visual differences. Further studies are ongoing to evaluate the optimal filter settings for PURE. Additionally, clinical outcomes were not within the scope of this study and responses were only gathered from three blinded reviewers. Lastly, not all mapping and recording systems currently available in the market were evaluated in this study.

## 5 CONCLUSION

The PURE intracardiac signals were statistically rated better than conventional systems on overall signal quality, confidence in discerning NF versus FF signal components, and the clinical significance of small and/or fractionated signals. Access to advanced signal processing technology could potentially improve ablation efficiency and clinical outcomes but further studies are needed to validate this hypothesis.

## Supporting information

Supplementary information.

## Data Availability

The data that support the findings of this study are available from the corresponding author upon reasonable request.

## References

[1] Josephson ME , Scharf DL , Kastor JA , Kitchen JG . Atrial endocardial activation in man. Electrode catheter technique of endocardial mapping. Am J Cardiol. 1977;39(7):972‐981. 10.1161/01.cir.51.5.786, https://www.ncbi.nlm.nih.gov/pubmed/1122581 141203

[2] Kastor JA , Goldreyer BN , Moore EN , Shelburne JC , Manchester JH . Intraventricular conduction in man studied with an endocardial electrode catheter mapping technique. Patients with normal QRS and right bundle branch block. Circulation. 1975;51(5):786‐796. 10.1161/01.cir.51.5.786, https://www.ncbi.nlm.nih.gov/pubmed/1122581 1122581

[3] Caracta AR , Damato AN , Gallagher JJ , et al. Electrophysiologic studies in the syndrome of short P‐R interval, normal QRS complex. Am J Cardiol. 1973;31(2):245‐253. 10.1016/0002-9149(73)91037-0, https://www.ncbi.nlm.nih.gov/pubmed/4686123 4686123

[4] Buxton AE , Josephson ME . Role of electrophysiologic studies in identifying arrhythmogenic properties of antiarrhythmic drugs. Circulation. 1986;73(2 Pt 2):II67‐II72. https://www.ncbi.nlm.nih.gov/pubmed/3943175 3943175

[5] Scheinman MA . Reflections on the first catheter ablation of the atrioventricular junction. Pacing Clin Electrophysiol. 2003;26(12):2315‐2316. 10.1111/j.1540-8159.2003.00366.x, https://www.ncbi.nlm.nih.gov/pubmed/14675019 14675019

[6] Albert CM , Stevenson WG . The future of arrhythmias and electrophysiology. Circulation. 2016;133(25):2687‐2696. 10.1161/CIRCULATIONAHA.116.023519, https://www.ncbi.nlm.nih.gov/pubmed/27324363 27324363 PMC4930113

[7] Ravelli F , Mase M . Computational mapping in atrial fibrillation: how the integration of signal‐derived maps may guide the localization of critical sources. Europace. 2014;16(5):714‐723. 10.1093/europace/eut376, https://www.ncbi.nlm.nih.gov/pubmed/24798961 24798961

[8] Venkatachalam KL , Herbrandson JE , Asirvatham SJ . Signals and signal processing for the electrophysiologist: part I: electrogram acquisition. Circ Arrhythm Electrophysiol. 2011;4(6):965‐973. 10.1161/CIRCEP.111.964304, https://www.ncbi.nlm.nih.gov/pubmed/22203661 22203661

[9] Venkatachalam KL , Herbrandson JE , Asirvatham SJ . Signals and signal processing for the electrophysiologist: part II: signal processing and artifact. Circ Arrhythm Electrophysiol. 2011;4(6):974‐981. 10.1161/CIRCEP.111.964973, https://www.ncbi.nlm.nih.gov/pubmed/22203662 22203662

[10] Padmanabhan D , Sugrue A , Vaidya V , et al. Incremental benefit of a novel signal recording system during mapping and ablation. Europace. 2021;23(1):130‐138. 10.1093/europace/euaa194, https://www.ncbi.nlm.nih.gov/pubmed/33094311 33094311

[11] Arentz T , von Rosenthal J , Blum T , et al. Feasibility and safety of pulmonary vein isolation using a new mapping and navigation system in patients with refractory atrial fibrillation. Circulation. 2003;108(20):2484‐2490. 10.1161/01.CIR.0000097118.75179.83, https://www.ncbi.nlm.nih.gov/pubmed/14581401 14581401

[12] Walters TE , Lee G , Spence S , Kalman JM . The effect of electrode density on the interpretation of atrial activation patterns in epicardial mapping of human persistent atrial fibrillation. Heart Rhythm. 2016;13(6):1215‐1220. 10.1016/j.hrthm.2016.01.030, https://www.ncbi.nlm.nih.gov/pubmed/26829116 26829116

