# Supplementary figures and images for: Evaluation of a novel cardiac signal processing system for electrophysiology procedures: The PURE EP 2.0 study
Source: J Cardiovasc Electrophysiol. 2021 Oct 1;32(11):2915–22. doi: 10.1111/jce.15250 (PMC9293197; doi:10.1111/jce.15250)

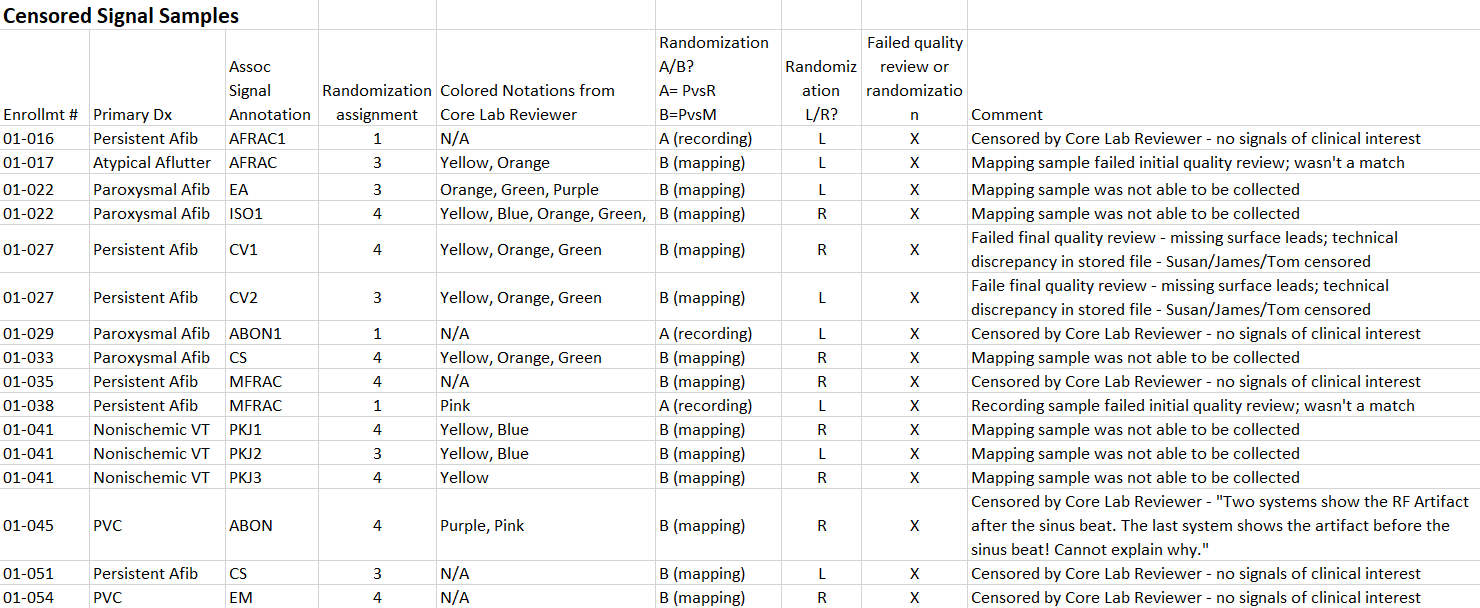


Supplement A

Supplement: Supplementary file 1 — Supplementary information. [file JCE-32-2915-s001.docx]
